# Chagas Disease and the London Declaration on Neglected Tropical Diseases

**DOI:** 10.1371/journal.pntd.0003219

**Published:** 2014-10-09

**Authors:** Rick L. Tarleton, Ricardo E. Gürtler, Julio A. Urbina, Janine Ramsey, Rodolfo Viotti

**Affiliations:** 1 Center for Tropical and Emerging Global Diseases, University of Georgia, Athens, Georgia, United States of America; 2 The Chagas Disease Foundation, Bogart, Georgia, United States of America; 3 Laboratory of Eco-Epidemiology, Department of Ecology, Genetics and Evolution, Universidad de Buenos Aires, Buenos Aires, Argentina; 4 Venezuelan Institute for Scientific Research, Caracas, Venezuela; 5 Centro Regional de Investigación en Salud Pública, Instituto Nacional de Salud Pública, Tapachula, Chiapas, México; 6 Hospital Interzonal General de Agudos (HIGA) Eva Perón, Sección Chagas, Servicio de Cardiología, Buenos Aires, Argentina; Universidad Autónoma de Yucatán, Mexico

## What Is Chagas Disease?

American trypanosomiasis is a chronic parasitosis caused by the kinetoplastid parasite *Trypanosoma cruzi* and is highly prevalent among a large variety of marsupial and placental mammals autochthonous to the American continent. The infection is naturally transmitted by blood-feeding Reduviid insects, but transmission by oral contamination, transplacentally, or by blood transfusion or issue transplantation is also common. The human disease is known as “Chagas disease” for the Brazilian physician who described it over a century ago. The human invasion of natural ecotopes as well as the establishment of the vectors in human dwellings associated with poor socioeconomic conditions makes Chagas disease a major public health hazard from the United States to Argentina. As such, the disease is a zoonosis that has afflicted humanity since its earliest presence in the New World and is still the largest parasitic disease burden on the American continent [1], [2]. Recently, increased international migrations have spread the infection to nonendemic areas, including Western Europe, Australia, and Japan, where transmission is restricted to congenital and transfusion or solid organ transplant. In most infected individuals, a highly effective immune response controls the initial infection but fails to eradicate it. The consequential lifelong infection and associated inflammatory response result in symptomatic cardiac and digestive disease, significant morbidity, and eventually death in 30%–40% of patients.

## Successes and Advances

Among the successes in the control and prevention of Chagas disease is the reduction of vector-based transmission in some countries in the Southern Cone of South America using a combination of widespread and recurrent domestic application of pyrethroid insecticides and screening of blood donations to prevent transfusion-related transmission. Chagas disease also benefits from having two nitro-heterocyclic drugs (benznidazole and nifurtimox) that have proven to be partially effective in use for >40 years in humans. The fact that *T. cruzi* infects many different mammal species is both a curse and blessing, as this parasite will never be eradicated and thus there will always be a risk of infection to humans. However, the wide host range of *T. cruzi* provides multiple excellent and highly relevant host models to evaluate immune responses and test specific treatments. This latter advantage is rare among neglected diseases. Furthermore, nonhuman host species that serve as links in the transmission to humans are being identified and can be targeted for control of transmission. The imminent completion of clinical trials to assess the benefit of treatment with benznidazole during chronic infection in humans should address a long-standing question regarding the clinical benefit of treating patients with long-established chronic *T. cruzi* infections [3]–[5]. In addition, human clinical trials of several new therapies are advancing or have been recently completed [5]–[7].

In addition to these operational advances, research developments over the last 20 or more years provide baseline information for improving detection, prevention, and control of *T. cruzi* infection. In contrast to long-held views on the autoimmune origin of the pathology of the chronic stage of Chagas disease, multiple lines of investigation confirm that the persistence of parasites is the key factor underlying the sustained inflammatory responses that lead to such manifestations [8], [9]. Thus, the condition should be treated as an infectious, not an autoimmune, disease, and specific treatment should be offered to all seropositive patients, perhaps with the exception of those with terminal disease [2], [10]. The challenges for sensitive serodiagnosis of *T. cruzi* infection and Chagas disease remain to be solved. The use of multiple, partially informative tests that ignore “discordant” samples (i.e., those positive on one but not all serologic tests) is not a sustainable and effective way to identify all those who need treatment. The mechanisms of action of existing treatments are only beginning to be understood [2], [11], and this provides hope that we can ultimately understand why treatment sometimes fails. Novel treatment regimens and combination therapies with currently available drugs, as well as drug candidates with novel mechanisms of action, are being preclinically [12]–[14] and clinically evaluated [5]–[7], [15]. Studies of the immune response to *T. cruzi* are identifying ways in which we may enhance parasite-specific immune responses [16] but are also raising questions about the potential for developing effective vaccines in the foreseeable future.

## What Are the 2020 London Declaration Goals for Chagas Disease?

The London Declaration on Neglected Tropical Diseases (http://www.unitingtocombatntds.org/) is an effort to eliminate or control ten neglected diseases by 2020—six years from now. Chagas disease is among these. The effort was launched in January 2012, spearheaded by the WHO and the Bill and Melinda Gates Foundation (BMGF) and including in the collaboration many of the world's leading pharmaceutical companies. To set specific goals and monitor achievement of those goals, the principals behind the London Declaration have established milestones for each disease (Table 1) and plan a yearly review and issuance of a “scorecard” of progress. For Chagas disease, the initial milestones were established primarily by WHO with rather limited input from the research community, at-risk communities, patient associations, or healthcare services within endemic countries. The purpose of the present document is to (1) assess these milestones and address if they can be achieved and if so, how; (2) propose additional milestones when appropriate and document other achievements to date towards these goals, and (3) identify the tools, infrastructure, and resources that are needed to achieve the overall goal of effective control of *T. cruzi* infection by the targeted 2020 date. Additional input on the London Declaration milestones (as well as a quick view of the current scorecard) can also be logged at https://sites.google.com/site/chagasddc/home/chagas-disease-milestones.

**Table 1:** London Declaration: WHO proposed milestones for 2020.

100% screening of tranfusional transmission
100% of countries certified with no intradomiciliary transmission in Latin America
100% of countries certified with no vectorial domiciliary infestation in Southeast Asia and the Western Pacific
100% of countries with access to antiparasitic treatment
100% of countries with certification of organ transplantation interruption
100% of countries with certification of transfusional transmission interrupted
100% of countries with control of congenital transmission
100% of Latin American countries with a surveillance system and prevention measures for oral transmission
100% of infected/ill patients under care
Domiciliary transmission interrupted in the region of the Americas

## Current Status of Chagas Disease

1. The full magnitude of the problem is only a guess because screening for infection is inconsistently applied, especially in endemic, resource-constrained rural areas in which infection prevalence has been historically high—“seek, and ye shall find.”
2. Current diagnostics probably identify the majority but certainly not all infected individuals, even when the standard two or three distinct testing platforms are used.
3. Vector transmission of *T. cruzi* has been interrupted only in some countries or regions of the Southern Cone. The sustainability of that achievement and its extension to other endemic areas is compromised by, among other factors, persisting infestations after routine insecticide spraying, abundance of other vector species, limited resources, lack of sustained political will, decentralization of disease control programs, dwindling numbers of trained personnel in charge of vector control operations, and the ever-increasing expansion of dengue outbreaks [17]–[19]. Recent documentation in northern Argentina and Bolivia of increasing numbers of foci of vector species highly resistant to the frontline pyrethroids may make current vector control protocols useless in some of the regions of highest transmission [20]–[22]. New and better insecticides are not in the pipeline. Little is known about the impact of the dramatic changes in the demographic and ecological landscape of Latin America on the extent and modes of *T. cruzi* transmission to humans. It is also becoming increasingly clear that transmission of *T. cruzi* is not limited to rural communities; urban and periurban vector-mediated transmission has been documented in Peru, Bolivia, Argentina, Mexico, and Venezuela, among others [23], [24].
4. Although reports of *T. cruzi* transmission unrelated to “conventional” vector infestation of houses, including via contaminated food or drink, congenital transmission, and by blood transfusion and tissue transplantation, have increased in recent decades [25], [26], and the extent and impact of these transmission modes has not been fully assessed in all affected countries.
5. Although treatment of the infection using currently available drugs can be effective, this is not always the case [2], [27]. Treatment, when offered, is generally restricted to only certain age groups in urban settings, and it has recently been estimated that less than 1% of those currently living with *T. cruzi* infections have received treatment [28]. Rural populations display the highest levels of infection prevalence and the lowest treatment coverage rates—the quintessence of neglect and inequity. The lack of screening programs to identify those who are infected and the inaccessibility of drugs due to cost or inadequate supply greatly limit the number of infected individuals receiving treatment. However, perhaps the biggest bottleneck in getting treatment to affected individuals is the lack of knowledge among at-risk populations and health and healthcare personnel and thus a failure to seek or prescribe treatment because of lack of understanding of its benefits. Many of these issues are surmountable using resources and knowledge already available—e.g., the capacity to produce affordable drugs is relatively high, and adverse reactions to treatment can be managed so that most can complete treatment. Unfortunately, when treatment failures occur, they are difficult to detect due to the lack of validated biomarkers for infection control or parasitological cure [29]–[31].

## General Comments on the Current London Declaration Milestones for Chagas Disease

1. The wording of some of the milestones makes it difficult to understand what is being assessed and how. For example, “30% of countries with certification of organ transplantation interruption” presumably indicates that effective programs to prevent transmission of infection via organ transplantation are in place in 30% of countries.
2. Many terms are vague and require more specific definition. For example, what does being “certified” and having “certification” involve [32], [33], and what qualifies as “access to treatment” and “control of transmission?” What specific countries make up the “100%?”
3. Some milestones are addressing problems that are nonexistent, never existed, and are quite unlikely to emerge, e.g., “countries certified with no vectorial domiciliary infestation on Southeast Asia and the Western Pacific.” A more frequently cosmopolitan species, *Triatoma rubrofasciata*, has been identified in Vietnam and other locations, but no *T. cruzi* infection has been demonstrated so far.
4. There are severe conflicts of interest in setting the milestones and assessing their achievement, with no indication of how and by whom the achievement will be determined and with what data. The organizations leading the initiative have a natural vested interest in declaring, for example, that elimination of transmission has been achieved in every county, department, state, or country. The information used for certifying the interruption of transmission is provided by the same health authorities in charge of disease control programs, who are also eager to claim they have achieved elimination because of the political value it affords. Moreover, current methods and procedures used to assess transmission indices have severe limitations [33]. The pressure to claim success is enormous, and there is very little expertise and independence to actually determine that disease transmission has been interrupted anywhere.
  As a result, there is very little trust that the certification of met milestones will be believable or can endure the “curse of success” and prevent disease resurgence in the foreseeable future. The milestones also do not consider or recommend the information systems needed to be able to verify and validate information. Where there is a persistent problem, as in El Salvador, there is no mention. Some control programs have been shut down, as in Ecuador; others have been downscaled, as in Colombia; and disease control efforts are passive and rudimentary in countries such as Mexico. Much of the data in official documents have not been independently verified to justify or back up strategies, conclusions, and recommendations.
5. Perhaps most importantly, the milestones are based on the assumption that the programs, tools, and resources to achieve them are already in place. They are not. Where are the diagnostics, access to or a system for distribution of treatments, measurement of vector infestation, and the integrated bug control programs that are going to be needed to achieve these milestones? Expansion of treatment with benznidazole is moving very, very slowly in the most affected areas of the Southern Cone countries and much less so elsewhere, and it is not always a question of drug availability but rather lack of procurement procedures or political priority and social equity. Furthermore, the long-standing, widespread foci of *T. infestans* highly resistant to pyrethroids must be suppressed immediately, their fate monitored closely, and the results of these efforts made public. More than a decade after its discovery, there is no further information regarding this unique situation even though the opportunity for spread of these resistant vector populations is high.
6. Previous policy initiatives and documentation of successes in control or elimination of transmission in the regions are neither appropriately focused nor consistent with scientific data. For example, there has been a push to issue statements of elimination of native triatomine species that thrive in the wild and invade houses often, some of them infected, but that only occasionally transmit *T. cruzi* to humans [33]. Such initiatives allow declarations of so called “success” but have minimal impact on overall transmission.In short, by underestimating the magnitude of the actual problem, overestimating what has been achieved or is in place and effective, and failing to identify the actual challenges, the London Declaration and Scorecard continues to build the case that “we're moving fast and easily towards Chagas elimination by the year 2020.” The majority opinion in the scientific, medical, and public health communities is that this is not the case. The status of Chagas disease control is highly heterogeneous between and within regions and countries and even within a given province, department, or county.

## What Needs to Be Done to Reach the Goal of Control of Chagas Disease by 2020?

1. Assess and carefully document the current extent of the problem of Chagas disease throughout the Americas.
  - Develop diagnostic tests that allow for dependable and inexpensive screening for all patient groups and in all areas.
  - Implement screening programs that will identify all infected individuals, regardless of age or other demographic attributes, and establish comprehensive periodic (at least annual) screening in areas where transmission is possible or likely.
  - Develop information systems to inventory and track all screening, treatment, and vector control efforts at the local, state or province, national, and international levels.
2. Reduce transmission.
  - Maintain and expand the vector control infrastructure, manpower, and expertise in all areas where transmission is occurring. Provide more training opportunities for vector control personnel and achieve high-quality application of insecticides and rigorous evaluation of their impact.
  - Design and implement sustainable vector and transmission (including oral, congenital, and transfusion or transplantation) control programs and develop independent assessments to evaluate their efficacy.
  - Develop efficient and effective monitoring systems to evaluate vector interventions, including reservoir community evaluation and vector population dynamics.
  - Identify the most common vehicles for oral transmission (how common is oral infection in the household setting, depending on the endemic region?) and develop methods to identify and prevent such transmission (e.g., public health education, facile methods to test for contamination, etc.).
  - Institute uniform testing and treatment of adolescents prior to child-bearing years and women of reproductive age, thus reducing opportunities for vertical transmission.
  - Facilitate commercialization of high-quality assays for blood and solid organ screening and educate health care professionals to recognize the need to screen solid organ donors as potential sources of infection.
  - Combine vector control efforts with treatment and education programs to increase acceptability, coverage, and sustainability.
3. Increase access to safe and effective treatments.
  - Provide dependable and affordable access to supervised benznidazole and nifurtimox treatment in all endemic countries and regions.
  - Promote the goal of promptly diagnosing and treating all infected individuals.
  - Use insights gained from ongoing and completed clinical trials to modify treatment paradigms.
  - Develop a better understanding of how current treatments work and when and why they fail and use this information to improve treatment regimens.
  - Discover and develop more effective treatments by pairing pharmaceutical industry know-how and resources with continuing new understanding of parasite biology and make use of in vitro screening tools and rigorous in vivo models for testing drug efficacy. Evaluate drug combinations in experimental and human Chagas disease.
  - Develop the tools for accurate assessment of treatment success in humans. Patients and physicians need to know if a particular treatment has been effective. The implementation of new treatment regimens requires the ability to accurately compare the efficacy of treatment protocols in humans. At present, this is not possible.
4. Establish accessible and rigorously documented databases of diagnostic screening efforts, vector control operations and programs, and treatment programs so that progress towards the London Declaration goals can be effectively monitored and more easily quantified.
5. Integrate Chagas disease into clinical care systems in all countries using workshops, courses, and continuing education, and promote equal access to diagnosis and clinical care.

## Proposed Milestones for 2014–2015

The milestones for the short term need to focus on obtaining accurate information, developing sound policies, and assuring that insecticides, diagnostics, and drugs are readily available.

1. Develop consensus policies for obtaining reliable, representative surveys of infected people (with and without treatment), house infestation with target vector species, and coverage and effectiveness of control efforts in all affected countries.
2. Obtain commitments from health ministries from all endemic countries to implement disease and vector control policies that are developed in consultation with the scientific community, patient groups, and other nongovernmental organizations.
3. Investigate the means, including legislation if needed, to implement routine periodic diagnostic screening of all primary school-age children and all women of reproductive age living or who have lived in areas where transmission of *T. cruzi* is possible or likely.
4. Through input from scientists active in drug development and testing and clinicians with experience in treating patients, develop a consensus document on best practices for treating and monitoring treatment outcomes for those with *T. cruzi* infection.
5. Obtain an accurate assessment of availability of benznidazole and nifurtimox, including current commercial, government and nongovernmental stockpiles, country-by-country planned distribution of those stores, anticipated future needs, and the resources available and required to make treatment readily available to all infected individuals. Determine the country-by-country cost of treatment and the funds available and/or needed to cover these costs. Develop mechanisms to track drug delivery and usage from the federal stockpiles to the province, state, and municipal levels.
6. Convene a meeting of researchers, clinicians, industry representatives, and nongovernmental organizations to assess options for diagnosing *T. cruzi* infection and to develop proposals to improve the quality and lower the cost of screening and diagnostic assays for *T. cruzi* infection.
7. Develop a reliable assessment of the operational capacity of triatomine vector control programs (e.g., gear, vehicles, personnel, and insecticides), program strategies, where and how often control operations are conducted, and methods used to monitor their effectiveness.

## What Additional Advances Are Needed by 2020 If Chagas Disease Is to Be Eliminated as a Human Health Problem?

Appropriate investments and political commitment can translate into actions that will reduce the impact of Chagas disease significantly by 2020. As outlined above, this will require a combination of more efficient healthcare systems, information systems, infrastructure (for diagnostic screening, vector control, drug production and distribution, etc.), improved tools (e.g., diagnostic assays, methods to monitor treatment efficacy, etc.), and broader knowledge and a better dialogue with communities and their acceptance of and collaboration with these projects. Without these developments, it is virtually impossible that any of the proposed 2020 milestones for Chagas disease in the London Declaration can be met. Establishing these milestones without a plan to develop appropriate resources is a recipe for failure.

In addition to the resources needed to reduce the impact of Chagas disease by 2020 as described above, concurrently there have to be additional investments in research if Chagas disease is to be eliminated as a human health problem, including in the following areas:

1. Drug discovery and testing. There is currently substantial interest and ongoing efforts in drug discovery for Chagas disease. Unfortunately, many of these efforts are poorly organized, disconnected, and woefully underfunded. Drug discovery is often divorced from parasite biology and pharmacological and infection parameters, promising leads are not vigorously pursued, and existing drugs and clinical candidates are not rigorously tested using the best possible model systems. As a result, compounds are going to clinical trials with insufficient data—and then failing in those trials—and the pipeline for new clinical candidates is nearly empty. Virtually nothing is known about the mode of action of current drugs, and efforts to optimize dosing strategies for monotherapies and combination therapies are nearly nonexistent. This is all happening in a landscape of incredibly good animal models for testing, including many nonhuman primate species with naturally acquired infections. *T. cruzi* infection can be treated and cured at any stage of the infection/disease. It is inexcusable that more people are not treated and that more effective treatments are not being effectively pursued.
2. Vector ecology and strategies for their control. The insecticidal spraying of houses to control vector species has done more than any other method to reduce the impact of Chagas disease. However, a more integrated approach is necessary for sustainable prevention. There are a number of excellent complementary interventions required for transmission control (e.g., use of insecticide-treated bednets or netting, preventing bugs from feeding on animal reservoirs by use of insecticide-treated dog collars, and/or vaccination of potential reservoirs to reduce their ability to transmit) [34], [35]. Extensively testing these methods individually or in combination is likely to lead to improved, lower cost and more sustainable vector control protocols. Investment in this area of research has to be made if we expect to significantly and permanently reduce the incidence of human infection with *T. cruzi*.
3. Vaccine development. Prophylactic vaccines are the most cost-effective means to prevent many human infections. It is yet to be proven that *T. cruzi* infection is vaccine preventable in any host species. Nevertheless, efforts in this area need to continue if for no other reason than to determine if vaccines are likely to be part of the long-term strategy for prevention of *T. cruzi* infection or if we will have to rely on other transmission control tools and treatment when these controls fail.

In addition to these research needs, it is also essential to incorporate at-risk and clinically affected populations into Chagas disease public health and clinical care program design and implementation. Civil society should be an integral consultant or collaborator in any public or private initiative, and it is important to analyze governance issues related to integration of an effective Chagas disease program within existing healthcare systems.

## Final Thoughts

We believe that Chagas disease is a solvable problem. The London Declaration on Neglected Tropical Diseases initiative provides an enormous opportunity to implement solutions. This opportunity should not be squandered by having weakly vetted and ill-defined goals. We hope that the current document can serve as a blueprint that all the communities involved in and affected by Chagas disease can contribute to and rally around. We welcome comments and additional suggestions at https://sites.google.com/site/chagasddc/home/chagas-disease-milestones.

## References

[1] Hotez PJ, Bottazzi ME, Franco-Paredes C, Ault SK, Periago MR (2008) The neglected tropical diseases of latin america and the Caribbean: a review of disease burden and distribution and a roadmap for control and elimination. PLoS Negl Trop Dis 2: e300. 18820747 10.1371/journal.pntd.0000300 PMC2553488

[2] Urbina JA (2010) Specific chemotherapy of Chagas disease: relevance, current limitations and new approaches. Acta Trop 115: 55–68. 19900395 10.1016/j.actatropica.2009.10.023

[3] Marin-Neto JA, Rassi A Jr, Avezum A Jr, Mattos AC, Rassi A, et al (2009) The BENEFIT trial: testing the hypothesis that trypanocidal therapy is beneficial for patients with chronic Chagas heart disease. Mem Inst Oswaldo Cruz 104 Suppl 1: 319–324 BENEFIT: Evaluation of the Use of Antiparasital Drug (Benznidazole) in the Treatment of Chronic Chagas' Disease. Population Health Research Institute. Available: http://clinicaltrials.gov/. Identifier: NCT00123916. 19753491 10.1590/s0074-02762009000900042

[4] Riarte A (2013) TRAENA: Placebo-controlled evaluation of impact of benznidazole treatment on long-term disease progression in adults with chronic Chagas disease. In: Proceedings of the 62nd Annual Meeting of the American Society of Tropical Medicine and Hygiene; November 13–17, 2013; Washington, DC.

[5] Torrico F (2013) E1224–Results of proof of concept clinical trial in patients with chronic indeterminate Chagas disease. In: Proceedings of the 62nd Annual Meeting of the American Society of Tropical Medicine and Hygiene; November 13–17, 2013; Washington, DC. Proof-of-Concept Study of E1224 to Treat Adult Patients With Chagas Disease. Drugs for Neglected Diseases Initiative. Available: http://clinicaltrials.gov/. Identifier: NCT01489228.

[6] Molina I (2012) First clinical trial with posaconazole and benznidazole for the treatment of chronic Chagas disease. In: Proceedings of the XVIII International Congress of Tropical Medicine and Malaria; September 23–27, 2012; Rio de Janeiro, Brazil. Available: http://ictmm2012.ioc.fiocruz.br/program_25_sept.html. Accessed 8 September 2014. Clinical Trial For The Treatment Of Chronic Chagas Disease With Posaconazole And Benznidazole (CHAGASAZOL). Hospital Universitari Vall d'Hebron Research Institute. http://clinicaltrials.gov/. Identifier: NCT01162967.

[7] A Study of the Use of Oral Posaconazole (POS) in the Treatment of Asymptomatic Chronic Chagas Disease (P05267) (STOP CHAGAS). Merck Sharp & Dohme Corp. Available: http://clinicaltrials.gov/. Identifier: NCT01377480. Accessed 8 September 2014.

[8] Tarleton RL (2001) Parasite persistence in the aetiology of Chagas disease. Int J Parasitol 31: 549–553. 10.1016/s0020-7519(01)00158-8 11334941

[9] Tarleton RL (2003) Chagas Disease: a role for autoimmunity? Trends Parasitol 10: 447–451. 10.1016/j.pt.2003.08.008 14519582

[10] Viotti R, de Noya BA, Araujo-Jorge T, Grijalva MJ, Guhl F, et al (2014) Towards a paradigm shift in the treatment of chronic Chagas disease. Antimicrob Agents Chemother 58: 635–639. 24247135 10.1128/AAC.01662-13 PMC3910900

[11] Wilkinson SR, Bot C, Kelly JM, Hall BS (2011) Trypanocidal activity of nitroaromatic prodrugs: current treatments and future perspectives. Curr Top Med Chem 11: 2072–2084. 21619510 10.2174/156802611796575894

[12] Cencig S, Coltel N, Truyens C, Carlier Y (2012) Evaluation of benznidazole treatment combined with nifurtimox, posaconazole or AmBisome(R) in mice infected with Trypanosoma cruzi strains. Int J Antimicrob Agents 40: 527–532. 23063742 10.1016/j.ijantimicag.2012.08.002

[13] Diniz Lde F, Urbina JA, de Andrade IM, Mazzeti AL, Martins TA, et al (2013) Benznidazole and posaconazole in experimental Chagas disease: positive interaction in concomitant and sequential treatments. PLoS Negl Trop Dis 7: e2367. 23967360 10.1371/journal.pntd.0002367 PMC3744424

[14] Bustamante J, Craft JM, Ketchie SA, Tarleton RL (2014) Efficacy of alternate and combination drug treatment protocols in cure of *Trypanosoma cruzi* infection in mice. J Infect Dis 209: 150–162. 23945371 10.1093/infdis/jit420 PMC3864384

[15] Perez-Mazliah DE, Alvarez MG, Cooley G, Lococo BE, Bertocchi G, et al (2013) Sequential combined treatment with allopurinol and benznidazole in the chronic phase of Trypanosoma cruzi infection: a pilot study. J Antimicrob Chemother 68: 424–437. 23104493 10.1093/jac/dks390 PMC3543119

[16] Kurup SP, Tarleton RL (2013) Perpetual expression of PAMPs is necessary for optimal immune control and clearance of an otherwise persistent pathogen. Nat Commun 4: 2616. 24149620 10.1038/ncomms3616 PMC4161029

[17] Cecere MC, Vazquez-Prokopec GM, Ceballos LA, Boragno S, Zarate JE, et al (2013) Improved chemical control of Chagas disease vectors in the dry Chaco region. J Med Entomol 50: 394–403. 23540129 10.1603/me12109 PMC3773707

[18] Gurtler RE (2009) Sustainability of vector control strategies in the Gran Chaco region: current challenges and possible approaches. Mem Inst Oswaldo Cruz 104 Suppl 1: 52–59. 19753458 10.1590/s0074-02762009000900009 PMC3072747

[19] Yadón ZE, Gürtler RE, Tobar F, Medici AC (2007) Decentralization and Management of Communicable Disease Control in Latin America. Buenos Aires: Pan American Health Organization. Available: http://www.paho.org/English/ad/dpc/cd/res-descentralizacion.htm. Accessed 8 September 2014.

[20] Picollo MI, Vassena C, Santo Orihuela P, Barrios S, Zaidemberg M, et al (2005) High resistance to pyrethroid insecticides associated with ineffective field treatments in Triatoma infestans (Hemiptera: Reduviidae) from Northern Argentina. J Med Entomol 42: 637–642. 16119553 10.1093/jmedent/42.4.637

[21] Lardeux F, Depickere S, Duchon S, Chavez T (2010) Insecticide resistance of Triatoma infestans (Hemiptera, Reduviidae) vector of Chagas disease in Bolivia. Trop Med Int Health 15: 1037–1048. 20545921 10.1111/j.1365-3156.2010.02573.x

[22] Gurevitz JM, Gaspe MS, Enriquez GF, Vassena CV, Alvarado-Otegui JA, et al (2012) Unexpected failures to control Chagas Disease vectors with pyrethroid spraying in northern Argentina. J Med Entomol 49: 1379–1386. 23270166 10.1603/me11157 PMC3760256

[23] Levy MZ, Bowman NM, Kawai V, Waller LA, Cornejo del Carpio JG, et al (2006) Periurban Trypanosoma cruzi-infected Triatoma infestans, Arequipa, Peru. Emerg Infect Dis 12: 1345–1352. 17073082 10.3201/eid1209.051662 PMC3294737

[24] Medrano-Mercado N, Ugarte-Fernandez R, Butron V, Uber-Busek S, Guerra HL, et al (2008) Urban transmission of Chagas disease in Cochabamba, Bolivia. Mem Inst Oswaldo Cruz 103: 423–430. 18797753 10.1590/s0074-02762008000500003

[25] Munoz-Calderon A, Diaz-Bello Z, Valladares B, Noya O, Lopez MC, et al (2013) Oral transmission of Chagas disease: typing of Trypanosoma cruzi from five outbreaks occurred in Venezuela shows multiclonal and common infections in patients, vectors and reservoirs. Infect Genet Evol 17: 113–122. 23567816 10.1016/j.meegid.2013.03.036

[26] Shikanai-Yasuda MA, Carvalho NB (2012) Oral transmission of Chagas disease. Clin Infect Dis 54: 845–852. 22238161 10.1093/cid/cir956

[27] Rassi A Jr, Dias JC, Marin-Neto JA, Rassi A (2009) Challenges and opportunities for primary, secondary, and tertiary prevention of Chagas' disease. Heart 95: 524–534. 19131444 10.1136/hrt.2008.159624

[28] Ribeiro I, Sevcsik AM, Alves F, Diap G, Don R, et al (2009) New, improved treatments for Chagas disease: from the R&D pipeline to the patients. PLoS Negl Trop Dis 3: e484. 19582163 10.1371/journal.pntd.0000484 PMC2702098

[29] Laucella SA, Perez Mazliah D, Bertocchi G, Alvarez MG, Cooley G, et al (2009) Changes in Trypanosoma cruzi-specific immune responses following treatment: surrogate markers of treatment efficacy. Clin Infect Dis 49: 1675–1684. 19877967 10.1086/648072 PMC2805187

[30] Cooley G, Etheridge RD, Boehlke C, Bundy B, Weatherly DB, et al (2008) High Throughput Selection of Effective Serodiagnostics for Trypanosoma cruzi infection. PLoS Negl Trop Dis 2: e316. 18841200 10.1371/journal.pntd.0000316 PMC2556098

[31] Fernandez-Villegas A, Pinazo MJ, Maranon C, Thomas MC, Posada E, et al (2011) Short-term follow-up of chagasic patients after benzonidazole treatment using multiple serological markers. BMC Infect Dis 11: 206. 21801456 10.1186/1471-2334-11-206 PMC3169482

[32] Abad-Franch F, Diotaiuti L, Gurgel-Goncalves R, Gurtler RE (2013) Certifying the interruption of Chagas disease transmission by native vectors: cui bono? Mem Inst Oswaldo Cruz 108: 251–254. 23579810 10.1590/0074-0276108022013022 PMC3970656

[33] Abad-Franch FDL, Gurgel-Gonçalvez R, Gürtler RE (2014) On bugs and bias: improving Chagas disease control assessment. Mem Inst Oswaldo Cruz 109: 125–130. 24809110

[34] Reithinger R, Ceballos L, Stariolo R, Davies CR, Gurtler RE (2006) Extinction of experimental Triatoma infestans populations following continuous exposure to dogs wearing deltamethrin-treated collars. Am J Trop Med Hyg 74: 766–771. 16687678 PMC2633875

[35] Levy MZ, Quispe-Machaca VR, Ylla-Velasquez JL, Waller LA, Richards JM, et al Impregnated netting slows infestation by Triatoma infestans. Am J Trop Med Hyg 79: 528–534. 18840739 PMC2659296

